# Mulberry (*Morus alba* L.) Fruit Extract Ameliorates Inflammation via Regulating MicroRNA-21/132/143 Expression and Increases the Skeletal Muscle Mitochondrial Content and AMPK/SIRT Activities

**DOI:** 10.3390/antiox10091453

**Published:** 2021-09-13

**Authors:** Sunyoon Jung, Mak-Soon Lee, Eugene Chang, Chong-Tai Kim, Yangha Kim

**Affiliations:** 1 Department of Nutritional Science and Food Management, Ewha Womans University, Seoul 03760, Korea; syjung583@dankook.ac.kr (S.J.); troph@hanmail.net (M.-S.L.); 2 Department of Food and Nutrition, Gangneung-Wonju National University, Gangneung 25457, Korea; echang@gwnu.ac.kr; 3 R&D Center, EastHill Corporation, Suwon 16642, Korea; ctkim@ieasthill.com; 4 Graduate Program in System Health Science and Engineering, Ewha Womans University, Seoul 03760, Korea

**Keywords:** mulberry fruit, inflammation, microRNA, skeletal muscle mitochondria, AMPK/SIRT, high-fat diet

## Abstract

The Mulberry (*Morus alba* L.) fruit is a rich source of polyphenolic compounds; most of these are anthocyanins. Obesity is intimately related to low-grade inflammation, with increased pro-inflammatory cytokine secretion and macrophage infiltration in white adipose tissue (WAT). This study investigated whether mulberry fruit extract (ME) has beneficial effects on obesity-induced inflammation and skeletal muscle mitochondrial dysfunction. Sprague-Dawley rats were divided into four groups and fed either a low-fat diet (LFD), high-fat diet (HFD), HFD + 5 g/kg of ME (ME-L), or HFD + 10 g/kg of ME (ME-H) for 14 weeks. ME alleviated dyslipidemia and lipid accumulation, as well as pro-inflammatory cytokine production such as tumor necrosis factor-α (TNF-α), interleukin 6 (IL-6), and monocyte chemoattractant protein 1 (MCP1) in the WAT. ME mitigated nuclear factor kappa-light-chain-enhancer of activated B cells (NF-κB) phosphorylation and macrophage infiltration in WAT. Notably, microRNA (miR)-21, miR-132, and miR-43 expressions were downregulated in the WAT of the ME groups compared to the HFD group. Moreover, ME increased the mitochondrial size and mitochondrial DNA (mtDNA) content, as well as key genes’ expression related to mitochondrial function, including sirtuin (SIRT)1, peroxisome proliferator-activated receptor γ coactivator-1α (PGC-1α), carnitine palmitoyltransferase 1β (CPT-1β), and uncoupling protein 3 (UCP3), and adenosine monophosphate-activated protein kinase (AMPK)/SIRT activities in skeletal muscle. These results suggested that ME might alleviate obesity-induced inflammation and mitochondrial dysfunction by regulating miR-21, miR-132, and miR-43 expression in WAT, and by activating the PGC-1α/SIRT1 pathway in muscle.

## 1. Introduction

Obesity and its-related consequence of oxidative stress contribute to the development of other metabolic diseases such as peripheral vascular disease, type 2 diabetes, and liver failure [1]. Inflammation is a manifestation of elevated oxidative stress in obesity [1]. Studies have reported that adipose tissue-derived inflammatory molecules mediate endothelial dysfunction and insulin resistance [1]. Hypertrophic adipocytes increase the production of pro-inflammatory cytokines, including tumor necrosis factor-α (TNF-α), interleukin 6 (IL-6), and monocyte chemoattractant protein 1 (MCP1) [2]. It has been reported that in an obese state, activated nuclear factor kappa-light-chain-enhancer of activated B cells (NF-κB) promotes the transcription of inflammatory mediators contributing to adipose tissue inflammation [2]. MicroRNA (miR) is a small non-coding RNA modulating gene expression at the post-transcriptional level. Several miRs, including miR-21, miR-132, and miR-143, have been reported to regulate adipogenesis and inflammation in adipocytes [3].

Obesity and inflammation are reciprocally related to impaired skeletal muscle function [4]. The skeletal muscle of obese subjects has lower mitochondrial content and incomplete fatty acid oxidation [5]. In addition, nutrient overload has been reported to promote mitochondria to produce excess reactive oxygen species (ROS) within cells, activating inflammatory signaling pathways and mitochondrial dysregulation [6]. In a systematic review, it has been reported that circulating inflammatory mediators, such as IL-6 and TNFα, are associated with lower skeletal muscle strength and muscle mass [7]. Chronic systemic inflammation has been proposed to contribute to skeletal muscle dysfunction through mitochondrial derangement [8]. Adenosine monophosphate (AMP)-activated protein kinase (AMPK) and sirtuin (SIRT) are key enzymes that regulate mitochondrial homeostasis [9]. AMPK stimulates SIRT enzymes by increasing cellular nicotinamide adenine dinucleotide (NAD) levels, leading to the expression of SIRT downstream genes related to mitochondrial biogenesis and function [10].

The Mulberry (*Morus alba* L.) fruit is a rich source of polyphenolic compounds; most of these are anthocyanins [11]. The principal anthocyanins in mulberry fruit are cyanidin-3-glucoside and cyanidin-3-rutinoside [11]. Many studies have reported beneficial effects on disease prevention of anthocyanins associated with antioxidant and anti-inflammatory properties [12]. Furthermore, mulberry fruit has been reported to possess many biological activities such as hypolipidemic [13], hypoglycemic [14], antioxidant [15], and immunomodulatory effects [16]. In particular, mulberry fruit was shown to inhibit fat accumulation and inflammation in obese animals [17,18]. Nevertheless, it is unclear whether the mulberry fruit improves obesity-induced adipose tissue inflammation and skeletal muscle mitochondrial function.

High hydrostatic pressure (HHP)-processing is a food processing technology that applies a high pressure above 0.1–500 Mpa at a temperature of 25 to 60 °C for the microbial inactivation and bioactive chemical compound extraction of foods [19]. HHP makes cell membranes of plants become more fragile, allowing plant bioactive compounds to elute solvents more easily [19]. Furthermore, as HHP-processing is performed at lower temperatures than conventional thermal processing, heat-sensitive bioactive compounds could be well preserved [19]. Indeed, in a study by Wang et al., the treatment of mulberry fruit juice with HHP significantly reduced the number of microorganisms, while phenolic compounds and antioxidant activities were better preserved than in heat-sterilized mulberry juice [20]. To better preserve the bioactive compounds of the mulberry fruit, in the preset study, HHP was applied during the extraction process.

Therefore, this study aimed to investigate the beneficial effects of HHP-treated mulberry fruit extract on adipose tissue inflammation and skeletal muscle mitochondrial function in rats fed a high-fat diet. To elucidate the molecular mechanisms by which mulberry fruit extract affects adipose tissue and skeletal muscle, for the first time, we analyzed adipose tissue miR expression and skeletal muscle AMPK/SIRT activities.

## 2. Materials and Methods

### 2.1. Mulberry Fruit Extract

The mulberry fruit extract (ME) was prepared by using a combination of HHP (100 MPa, 50 °C) and enzymatic hydrolysis with pectinase and was kindly supplied by the Korea Food Research Institute (Wanju, Korea). The preparation method of the ME has been previously described by Jung et al. [21]. The schematic illustration of the preparation method is presented in Supplementary Figure S1. The content of the total anthocyanin of the ME was 127.15 mg/100 g and consisted of cyanidin 3-O-glucoside (75.85 mg/100 g), cyanidin 3-O-rutinoside (49.08 mg/100 g), and pelargonidin 3-O-glucoside (2.22 mg/100 g) [21]. In addition, the total flavonol content of ME was 26.79 mg/100 g and mainly consisted of quercetin (12.99 mg/100 g), rutin (8.05 mg/100 g), and isoquercitrin (3.14 mg/100 g) [21].

### 2.2. Animals and Diets

All experimental procedures were approved by the Institutional Animal Care and Use Committee of Ewha Womans University, Korea (permission number 17-004). Male Sprague-Dawley rats (3 weeks old, weighing 43 ± 3 g) were purchased from Dooyeol Biotech (Seoul, Korea). The rats were housed individually in stainless-steel wire mesh cages under a controlled environment (a 12-h light/dark cycle, constant temperature of 22 ± 2 °C, and 55 ± 5% humidity). After four days of adaptation, rats were randomly divided into four groups and fed an experimental diet for 14 weeks as follows: (1) low-fat diet (LFD, 10% calories from fat), (2) high-fat diet (HFD, 45% calories from fat), (3) HFD supplemented with 5 g/kg diet of ME (ME-L), and (4) HFD supplemented with 10 g/kg diet of ME (ME-H). The compositions of the experimental diets are presented in Supplementary Table S1. The rats had free access to water and food. Their body weights and food intakes were measured once a week. At the end of the experiment, rats were anesthetized by intraperitoneal injection of a 5:2 (*v/v*) mixture of Zoletil 50^®^ (Virbac Laboratories, Carros, France) and Rompun^®^ (Bayer, Leverkusen, Germany) at a dose of 0.14 mL/100 g body weight following overnight fasting. After blood collection by cardiac puncture, the serum was separated by centrifugation (1300× *g*, 10 min) and stored at −70 °C until analysis. Liver and epididymal adipose tissue were excised, weighed, immediately frozen in liquid nitrogen, and kept at −70 °C until analysis.

### 2.3. Serum Biochemical Measurements

The serum levels of triglyceride (TG), total cholesterol (TC), high-density lipoprotein-cholesterol (HDL-C), aspartate transaminase (AST), and alanine transaminase (ALT) were determined using commercially available kits (Embiel, Gunpo, Korea). The value of low-density lipoprotein-cholesterol (LDL-C) was calculated by the Friedewald formula: LDL-C (mmol/L) = TC − HDL-C − (TG/2.2) [22]. The atherogenic index (AI) was calculated using the following formula: AI = (TC − HDL-C)/HDL-C. The serum nitric oxide (NO) level was measured using a Griess Reagent kit (Invitrogen, Carlsbad, CA, USA) following the manufacturer’s instructions.

### 2.4. Hepatic Lipid Analysis

Hepatic total lipids were extracted using the Bligh and Dyer method [23], with slight modification. Briefly, the liver sample (~0.3 g) was homogenized in 1 mL of 0.9% saline and then the homogenate was mixed with 3.8 mL of chloroform/methanol (1:2, *v/v*). After the addition of chloroform (1.25 mL) and distilled water (1.25 mL), the solution was thoroughly mixed and centrifuged at 1800× *g* for 5 min. Next, the lower clear organic phase solution was transferred into a new tube and then filtered through Whatman No. 6 filter paper (Whatman International Ltd., Maidstone, UK). The lipid in this phase was dried until a constant weight was reached. Finally, the lipid was dissolved in 2 mL of n-hexane/isopropanol (3:2, *v/v*) and stored at −20 °C until analysis. The hepatic concentrations of TG and TC were determined by commercial kits (Embiel) according to the manufacturer’s instructions.

### 2.5. Hematoxylin and Eosin (H&E) Staining

Epididymal adipose tissue was fixed in 10% neutral buffered formalin overnight at room temperature (RT; 20–25 °C). The fixed tissue was processed on a Leica TP 1020 automated tissue processor (Leica Microsystems, Wetzlar, Germany) as follows: incubation at 70%, 80%, 90%, and 100% ethanol (1 h × 2); then xylene (1 h × 2); and followed by paraffin (1 h × 2) under vacuum at 56 °C. The processed tissue was then embedded with paraffin and cut to a thickness of 6 μm in a microtome (Leica Microsystems). The paraffin sections of the tissue were stained with H&E. Digital images of sections were acquired using an Olympus IX51 inverted microscope (magnification, 200×; Olympus, Tokyo, Japan). The adipocyte size was analyzed using the ImageJ software (National Institutes of Health, Bethesda, MD, USA; https://imagej.nih.gov/ij/, accessed on 30 January 2018).

### 2.6. Immunohistochemistry (IHC)

Paraffin sections of epidydimal adipose tissue were prepared as described above and incubated with the anti-F4/80 (EGF-like module-containing, mucin-like, hormone receptor-like 1) antibody (GeneTex, Irvine, CA, USA). The antibodies were detected using the Polink-2 Plus HRP Anti-Rat DAB Detection kit (Golden Bridge International Inc., Mukilteo, WA, USA) according to the manufacturer’s instructions. The tissue sections were then counterstained with Mayer’s hematoxylin (ScyTek, West Logan, UT, USA). Digital images of stained sections were acquired using an Olympus IX51 inverted microscope (magnification, 200×; Olympus).

### 2.7. Quantitative Real-Time Polymerase Chain Reaction (qRT-PCR)

Total RNA from tissue was extracted using a RiboEx total RNA isolation solution (GeneAll Biotechnology, Seoul, Korea). Then, the RNA was reverse transcribed into cDNA using a M-MLV (Moloney Murine Leukemia Virus)-reverse transcriptase kit (Bioneer Co., Daejeon, Korea). The SYBR Green Mix (AccuPower 2X Greenstar qPCR MasterMix, Bioneer Co.) and Rotor-Gene 3000 (Corbett Research, Sydney, Australia) were used for the qRT-PCR. β-actin was used as an internal control to normalize gene expression. The primer sequences are listed in Supplementary Table S2.

For miR expression analysis, cDNA was synthesized using the ABM miRNA cDNA Synthesis Kit with Poly (A) Polymerase Tailing (ABM Inc., Richmond, BC, Canada). The cDNA was amplified using primers specific for miR-21, miR-132, miR-143, and RNU6B (ABM Inc.) with the EvaGreen miRNA qPCR Master Mix (ABM Inc.). The expression of each miRNA was normalized to that of RU6 snRNA.

The relative fold change of mRNA or miR expression was calculated using the delta-delta Ct method [24].

### 2.8. Western Blot Analysis

Total protein was extracted with an ice-cold RIPA buffer (Elpis Biotech, Daejeon, Korea) containing protease inhibitors (Roche Applied Sciences, Indianapolis, IN, USA) and phosphatase inhibitors (Roche Applied Sciences). Nuclear protein was extracted using an EpiSeeker Nuclear Extraction kit (Abcam, Cambridge, United Kingdom) according to the manufacturer’s instructions. Proteins were mixed with sample buffer (Laemmli’s 5×, Elpis Biotech) and boiled at 95 °C for 5 min. Equal amounts of the denatured proteins were subjected to 8% or 12% sodium dodecyl sulfate-polyacrylamide gel electrophoresis (SDS-PAGE) and transferred to polyvinylidene difluoride (PVDF) membranes using wet transfer equipment (Bio-Rad, Hercules, CA, USA). Next, the membranes were blocked using 5% non-fat milk in Tris-buffered saline with 0.1% Tween–20 (TBS-T) for 1 h at RT. Subsequently, the membranes were incubated with the primary antibody specific for F4/80 (1:500; GeneTex, Irvine, CA, USA), NF-κB p65 (1:2000; Abcam), β-actin (1:500; Bioss Antibodies, Woburn, MA, USA), or histone H3 (1:1000; Abcam) at 4 °C overnight. After being washed three times with 0.1% TBS-T, membranes were incubated with the horseradish peroxidase-conjugated secondary anti-rabbit antibody (1:4000; AbFrontier, Seoul, Korea) for 1 h at RT. Blots were then washed three times with TBS-T and immunoreactive protein was visualized using the PicoEPD Western Reagent (Elpis Biotech) and a Chemidoc XRS+ system (Bio-Rad). Band densities were quantified using ImageLab software (Bio-Rad). The densimetric values of the protein bands were normalized to that of β-actin and expressed as fold changes of the control group.

### 2.9. Enzyme-Linked Immunosorbent Assay (ELISA)

The levels of TNF-α, IL-6, MCP1, and NF-κB p65 were determined using commercially available ELISA kits. The TNF-α and IL-6 ELISA kits were purchased from BioLegend (San Diego, CA, USA). The MCP1 ELISA kit was purchased from Koma Biotech (Seoul, Korea) and the NF-κB p65 ELISA kit was obtained from Cayman (Ann Arbor, MI, USA). The values were expressed relative to the concentration of total protein. Total protein concentration was measured with a bicinchoninic acid (BCA) protein assay kit (Thermo Pierce Inc., Rockford, IL, USA).

### 2.10. Transmission Electron Microscopy (TEM)

Skeletal muscle tissue cut to within 2 mm was pre-fixed with 2% glutaraldehyde in 0.1 M phosphate buffer (pH 7.4) for 2 h and then post-fixed with 1% osmium tetroxide buffered by 0.1 M phosphate-buffered saline (PBS) (pH 7.4) for 1 h. Following dehydration with ethanol, the fixed tissue sample was embedded in epoxy resin (Epon 812) and cut into 1-μm thicknesses with an ultramicrotome (Reicher-Jung, Deerfield, IL, USA). After the tissue sections were stained with 1% toluidine blue, ultra-thin sections of interest of about 60 to 70 μm thick were cut and observed with an H-7650 transmission electron microscope (magnification 20,000×; Hitachi, Tokyo, Japan) at an accelerating voltage of 80 kV.

### 2.11. Mitochondrial DNA Content

Total DNA from skeletal muscle was extracted using a Puregene DNA isolation kit (Gentra Systems, Minneapolis, MN, USA) according to the manufacturer’s instructions. Relative mitochondrial DNA (mtDNA) content was determined by measuring the mitochondrial gene (Cox1, subunit 1 of cytochrome oxidase) relative to the nuclear gene (GAPDH, glyceraldehyde 3-phosphate dehydrogenase) using quantitative polymerase chain reaction (qPCR).

### 2.12. AMPK and SIRT Activities

AMPK activity from the total protein lysate was analyzed using an AMPK Kinase Assay kit (Cyclex, Nagano, Japan). SIRT activity from the nuclear protein was determined using a commercially available kit (Abcam, Cambridge, United Kingdom). Results were normalized to the protein concentration determined by a bicinchoninic acid (BCA) protein assay kit (Thermo Pierce Inc.) and expressed as fold changes of the control group.

### 2.13. Statistical Analysis

Statistical analyses were performed with SPSS (SPSS, version 19; IBM Corporation, Armonk, NY, USA). All data are expressed as means ± standard errors of the mean (SEMs). The differences among groups were determined by one-way analysis of variance (ANOVA), followed by Tukey’s post-hoc tests. A *p*-value less than 0.05 was considered statistically significant.

## 3. Results

### 3.1. Effects of ME on Body Weight, Food Intake, Liver Weight, and Serum AST and ALT Levels

After 14 weeks of the experiment, the final body weight and body weight gain of the HFD group were higher than those of the LFD group (*p* < 0.05; Table 1). Conversely, the ME-L and ME-H groups tended to have lower final body weights and body weight gains than the HFD group, although the differences were not statistically significant (Table 1). To evaluate whether the food consumption was related to the changes in the body weight, the rats’ daily food intakes, energy intakes, and food efficiency ratios (FER) were measured. The LFD group consumed less food than the HFD group (*p* < 0.05), whereas the food intakes among the HFD and ME-supplemented groups did not differ significantly (Table 1).

We further analyzed the liver weights and serum AST and ALT levels to determine whether the experimental diets affected hepatotoxicity. The liver weights and serum AST levels were not significantly different among the groups (Table 1). In contrast, the serum ALT level of the HFD group was significantly higher than that of the LFD group (*p* < 0.05); however, the serum ALT level in the ME-H group was considerably lower compared to that of the HFD group (*p* < 0.05; Table 1).

### 3.2. Effects of ME on Lipid Profiles of Serum and Liver

The serum TG levels in the ME-L and ME-H groups were significantly lower than that of the HFD group (*p* < 0.05; Table 2). HFD increased the serum TC level compared to the LFD group (*p* < 0.05); however, serum TC levels in the ME-L and ME-H groups were significantly lower than that in the HFD group (*p* < 0.05; Table 2). The levels of serum HDL-C and LDL-C did not significantly differ between the LFD and HFD groups, whereas the ME-H group had a higher HDL-C level and lower LDL-C level than the HFD group (*p* < 0.05; Table 2). Consequently, the AI value of the HFD group was significantly higher than that of the LFD group (*p* < 0.05); however, the ME-L and ME-H groups had substantially lower AI values compared to the HFD group (*p* < 0.05; Table 2).

In the livers of the HFD group, the levels of total lipid, TG, and TC were significantly higher than those of the LFD group (*p* < 0.05; Table 2). However, the hepatic levels of total lipid and TG in the ME-H group were significantly lower compared to those of the HFD group (*p* < 0.05; Table 2). Moreover, in both the ME-L and ME-H groups, the hepatic TC levels were significantly lower than in the HFD group (*p* < 0.05; Table 2).

### 3.3. Effects of ME on Adiposity

**Figure 1:**
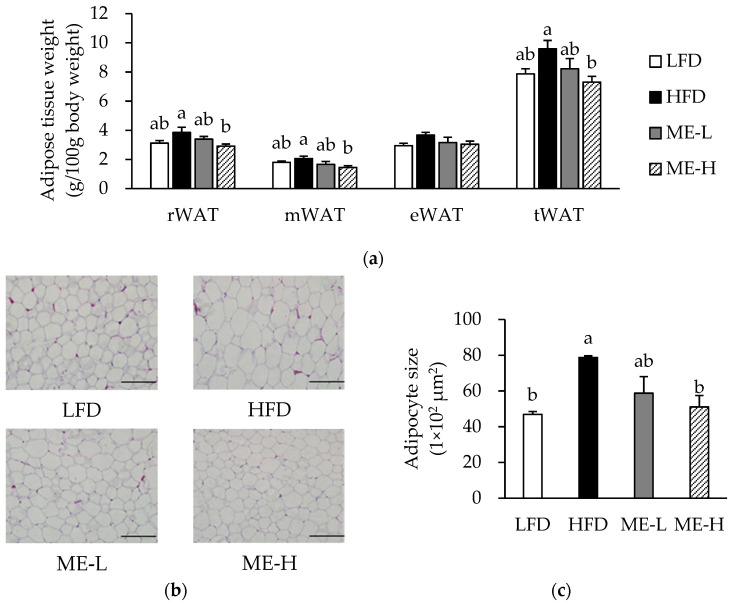

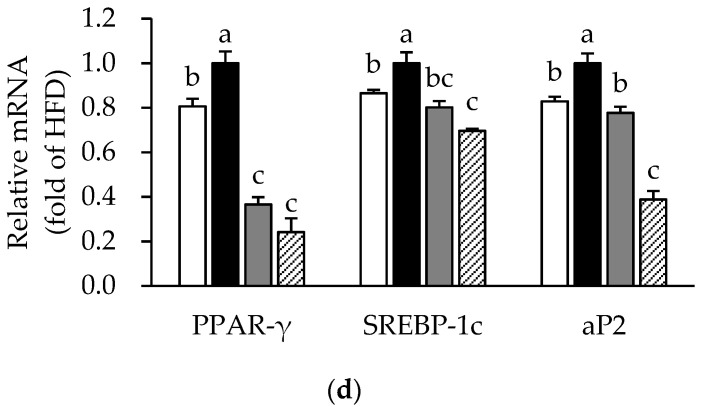
Effect of mulberry fruit extract on adiposity. (**a**) The relative weight of adipose tissue. (**b**) Representative images of hematoxylin and eosin (H&E)-stained sections of white adipose tissue. Magnification of 200. Scale bars = 200 μm. (**c**) Average adipocyte size. (**d**) mRNA expression of adipogenic genes in white adipose tissue. The mRNA levels were normalized relative to the β-actin. Values are expressed as mean ± SEM (n = 8/group). Bars with different letters indicate a significant difference at *p* < 0.05. Abbreviations: LFD, low-fat diet; HFD, high-fat diet; ME-L, HFD + 5 g/kg diet of mulberry fruit extract; ME-H, HFD + 10 g/kg diet of mulberry fruit extract; rWAT; retroperitoneal white adipose tissue; mWAT, mesenteric white adipose tissue; eWAT, epididymal white adipose tissue; tWAT, total white adipose tissue; PPAR-γ, peroxisome proliferator-activated receptor-γ; SREBP-1c, sterol regulatory element-binding protein-1c; and aP2, adipocyte protein 2.

In the HFD group, the weights of the retroperitoneal white adipose tissue (rWAT), mesenteric white adipose tissue (rWAT), epididymal white adipose tissue (rWAT), and total white adipose tissue (tWAT) tended to be higher than those in the LFD group (Figure 1a). In contrast, the ME-H group showed lower weights of rWAT, mWAT, and tWAT compared to the HFD group (*p* < 0.05; Figure 1a). The average adipocyte size of the HFD group was greater than that of the LFD group (*p* < 0.05); however, in the ME-H group, the adipocyte size was significantly smaller compared to the HFD group (*p* < 0.05; Figure 1b,c).

Since the ME inhibited adipocyte hypertrophy, we further analyzed the mRNA expression of genes related to adipogenesis in WAT. The mRNA expression levels of peroxisome proliferator-activated receptor (PPAR)-γ, sterol regulatory element-binding protein-1c (SREBP-1c), and adipocyte protein 2 (aP2) were significantly higher in the HFD group compared to the LFD group (*p* < 0.05); however, the mRNA expression levels of PPAR-γ, SREBP-1c, and aP2 in the ME-L and ME-H groups were significantly lower compared to those of the HFD group (*p* < 0.05; Figure 1d).

### 3.4. Effects of ME on Adipose Tissue Inflammation

**Figure 2:**
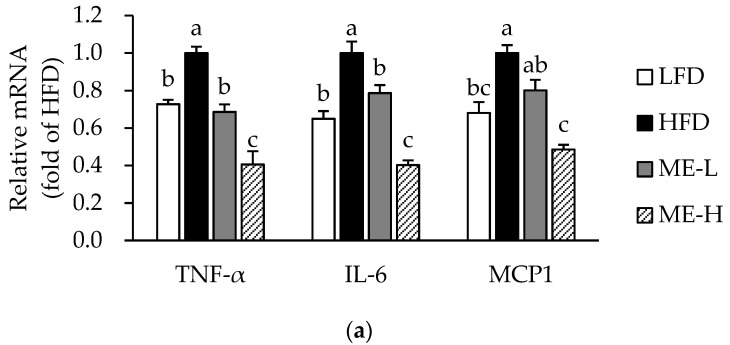

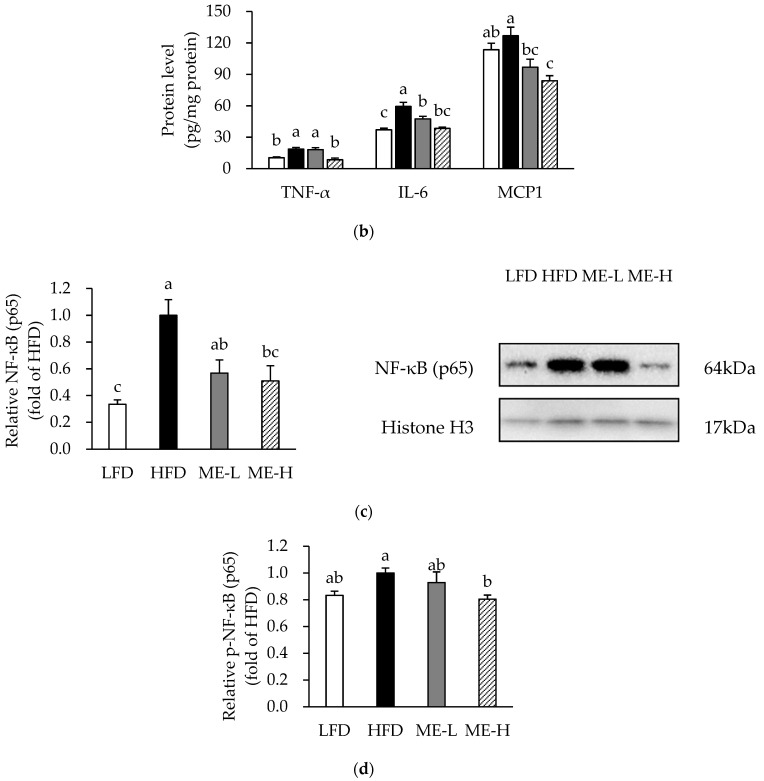
Effect of mulberry fruit extract on inflammation in white adipose tissue. (**a**) mRNA expression of pro-inflammatory cytokines. The mRNA levels were normalized relative to the β-actin. (**b**) Protein levels of pro-inflammatory cytokines. (**c**) Nuclear NF-κB p65 protein level. (**d**) Nuclear phosphorylated-NF-κB p65 level normalized to the total protein concentration. Values are expressed as mean ± SEM (n = 8/group). Bars with different letters indicate a significant difference at *p* < 0.05. Abbreviations: LFD, low-fat diet; HFD, high-fat diet; ME-L, HFD + 5 g/kg diet of mulberry fruit extract; ME-H, HFD + 10 g/kg diet of mulberry fruit extract; TNF-α, tumor necrosis factor-α; IL-6, interleukin 6; MCP1, monocyte chemoattractant protein 1; and NF-κB, nuclear factor-kappa B.

The mRNA expression levels of pro-inflammatory cytokines such as TNF-α, IL-6, and MCP1 were up-regulated in the HFD group compared to the LFD group (*p* < 0.05; Figure 2a). However, the mRNA expression levels of TNF-α and IL-6 were significantly lower in the ME-L and ME-H groups compared to the HFD group (*p* < 0.05), and the MCP1 mRNA expression was also lower in the ME-H group when compared to the HFD group (*p* < 0.05; Figure 2a). Figure 2b shows the protein levels of TNF-α, IL-6, and MCP1 in WAT. In the HFD group, the levels of TNF-α and IL-6 were significantly higher than in the LFD group (*p* < 0.05), and the MCP1 level also tended to be higher than in the LFD group (Figure 2b). However, the TNF-α level in the ME-H group was significantly lower than in the HFD group (*p* < 0.05) and both IL-6 and MCP1 levels were also lower in both the ME-L and ME-H groups compared to the HFD group (*p* < 0.05; Figure 2b).

To determine whether the anti-inflammatory effects of ME were associated with the regulation of the NF-κB axis, we further analyzed the protein levels of NF-κB (p65) and phosphorylated-NF-κB (p65) in nuclear extracts of WAT. As shown in Figure 2c, the nuclear NF-κB (p65) level of the HFD group was significantly higher than that of the LFD group (*p* < 0.05). In contrast, the level of NF-κB (p65) in the ME-H group was significantly lower compared to the HFD group (*p* < 0.05; Figure 2c). The nuclear phosphorylated NF-κB (p65) level tended to be higher in the HFD group than in the LFD group, but the level was significantly lower in the ME-H group compared to the HFD group (*p* < 0.05; Figure 2d).

### 3.5. Effects of ME on Macrophage Infiltration and Phenotypic Switching

**Figure 3:**
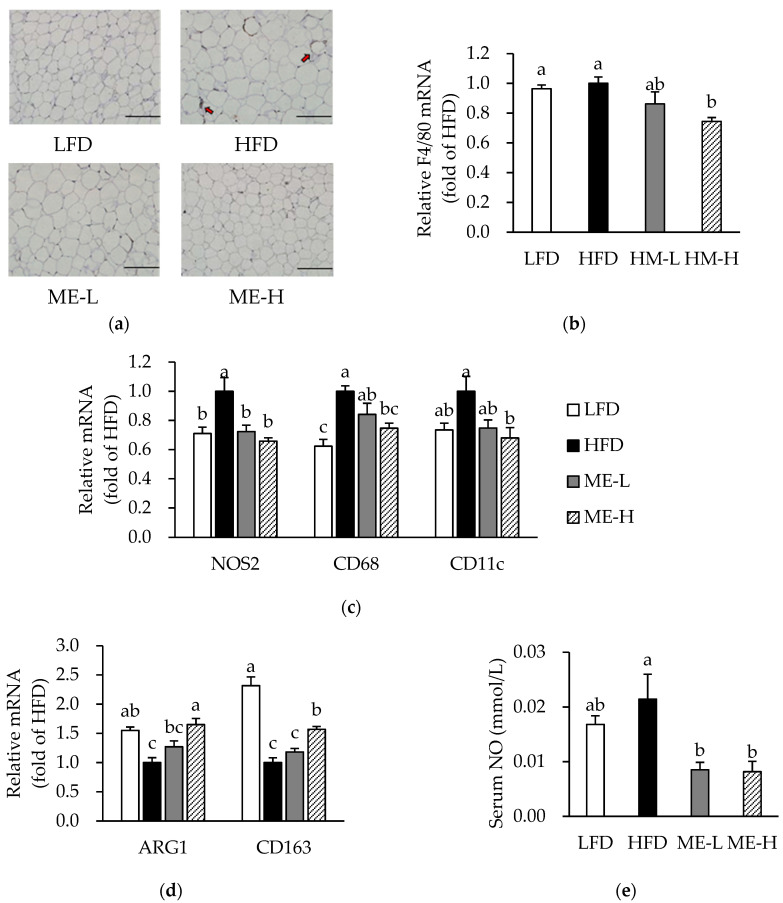
Effect of mulberry fruit extract on macrophage infiltration and M1/M2 type-switching in white adipose tissue. (**a**) Representative images of immunohistochemical staining for F4/80. Arrows indicate crown-like structures. Magnification of 200. Scale bars = 200 μm. (**b**) F4/80 mRNA expression. (**c**) mRNA expression of genes specific for M1 macrophages. (**d**) mRNA expression of genes specific for M2 macrophages. The mRNA levels were normalized relative to the β-actin. (**e**) Serum NO level. Values are expressed as mean ± SEM (n = 8/group). Bars with different letters indicate a significant difference at *p* < 0.05. Abbreviations: LFD, low-fat diet; HFD, high-fat diet; ME-L, HFD + 5 g/kg diet of mulberry fruit extract; ME-H, HFD + 10 g/kg diet of mulberry fruit extract; NOS2, nitric oxide synthase 2; CD, cluster of differentiation; ARG1, arginase 1; and NO, nitric oxide.

This study identified adipose tissue macrophage infiltration by IHC-staining and qRT-PCR analysis for F4/80. As shown in Figure 3a, macrophages in the HFD group formed a crown-like structure (CLS) surrounding adipocytes, but few CLSs were found in the other groups. The F4/80 mRNA levels did not differ significantly between the LFD and HFD groups; however, the F4/80 mRNA level of the ME-H group was lower than that of the HFD group (*p* < 0.05; Figure 3b).

As macrophages in obese adipose tissue promote phenotypic switching from M1 to M2 [25], we next analyzed mRNA expression levels specific for M1 and M2 macrophages, as well as for serum levels of NO. In the HFD group, the mRNA levels of nitric oxide synthase 2 (NOS2), cluster of differentiation (CD) 68, and CD11 were higher (*p* < 0.05), whereas the arginase 1 (ARG1) and CD163 mRNA levels were lower compared to the LFD group (*p* < 0.05; Figure 3c,d). In contrast, ME-H significantly suppressed the mRNA expression levels of NOS2, CD68, and CD11 while elevating ARG1 and CD163 mRNA levels (*p* < 0.05; Figure 3c,d). Meanwhile, the serum NO levels in both the ME-L and ME-H groups were significantly lower compared to the HFD group (*p* < 0.05; Figure 3e).

### 3.6. Effects of ME on MicroRNA Expression in WAT

**Figure 4:**
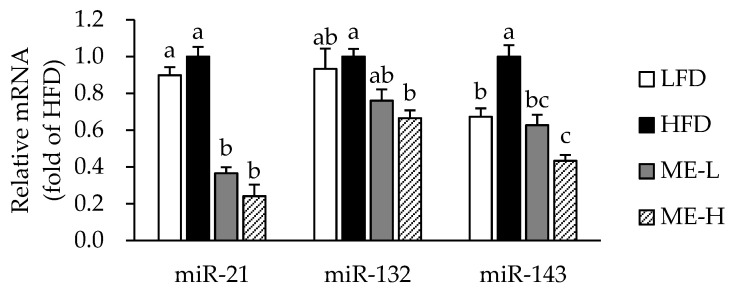
Effect of mulberry fruit extract on microRNA expression in white adipose tissue. The levels were normalized relative to the RNU6B. Values are expressed as mean ± SEM (n = 8/group). Bars with different letters indicate a significant difference at *p* < 0.05. Abbreviations: LFD, low-fat diet; HFD, high-fat diet; ME-L, HFD + 5 g/kg diet of mulberry fruit extract; ME-H, HFD + 10 g/kg diet of mulberry fruit extract; and miR, microRNA.

To investigate the molecular mechanism by which the ME alleviates adiposity and inflammation, we further analyzed miR-21, miR-132, and miR-143 in WAT. As shown in Figure 4, the expression of miR-143 was higher in the HFD group compared to the LFD group (*p* < 0.05); however, the expressions of miR-21 and miR-143 did not differ significantly between the LFD and HFD groups. Nevertheless, miR-21 and miR-143 expression levels were significantly lower in both the ME-L and ME-H groups than in the HFD group (*p* < 0.05). In addition, the miR-132 expression of the ME-H group was significantly lower than that of the HFD group (*p* < 0.05).

### 3.7. Effects of ME on Skeletal Muscle Mitochondrial Content and mRNA Expression Related to Mitochondrial Function

**Figure 5:**
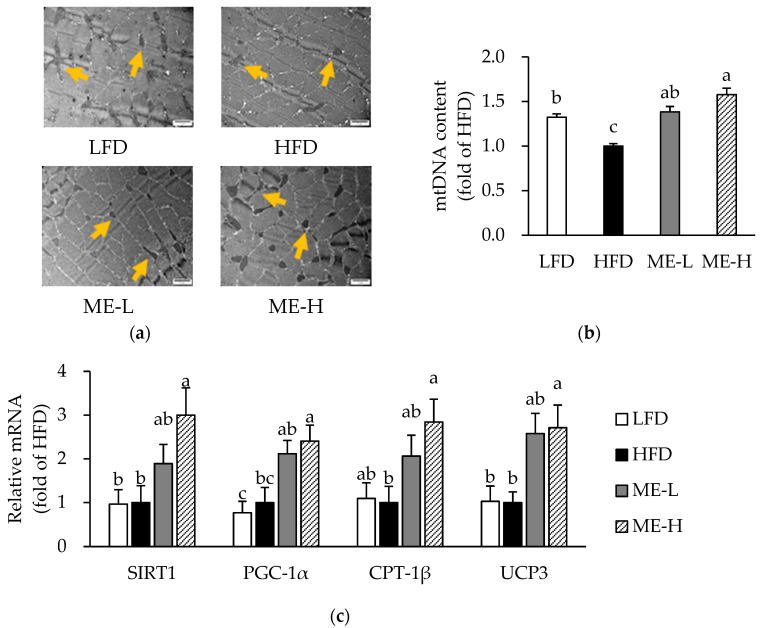
Effect of mulberry fruit extract on skeletal muscle mitochondria. (**a**) Representative transmission electron microscopic images. Magnification of 20,000. Scale bars = 2 μm. Arrows indicate mitochondria. (**b**) mtDNA content in skeletal muscle. The content was determined by measuring the mitochondrial gene, Cox1 (subunit 1 of cytochrome oxidase) relative to the nuclear gene, and GAPDH (glyceraldehyde 3-phosphate dehydrogenase). (**c**) mRNA expression of genes involved in mitochondrial function. The mRNA levels were normalized relative to the β-actin. Values are expressed as mean ± SEM (n = 8/group). Bars with different letters indicate a significant difference at *p* < 0.05. Abbreviations: LFD, low-fat diet; HFD, high-fat diet; ME-L, HFD + 5 g/kg diet of mulberry fruit extract; ME-H, HFD + 10 g/kg diet of mulberry fruit extract; and mtDNA, mitochondrial DNA.

Figure 5a shows the representative TEM images of skeletal muscle. In the HFD group, the number and size of mitochondria tended to be lower than in the LFD group; however, mitochondria in the ME groups appeared to be more numerous and larger in size than those of the HFD group. Therefore, we next analyzed mitochondrial mass, which reflects the number and size of mitochondria, by measuring the mtDNA content [26]. According to the qPCR analysis, the content of mtDNA in the HFD group was significantly lower than that in the LFD group; however, both the ME-L and ME-H groups had more mtDNA compared to the HFD group (*p* < 0.05; Figure 5b).

As ME affected mitochondrial morphology and number, we further analyzed the mRNA expression of genes involved in mitochondrial function in skeletal muscle. The mRNA levels of SIRT1, peroxisome proliferator-activated receptor-γ coactivator 1α (PGC-1), carnitine palmitoyltransferase 1β (CPT-1β), and uncoupling protein 3 (UCP3) were not significantly different between the LFD and HFD groups (Figure 5c). However, in the ME-H group, the mRNA levels of SIRT1, PGC-1α, CPT-1β, and UCP3 were significantly higher compared to the HFD group (*p* < 0.05; Figure 5c).

### 3.8. Effects of ME on Skeletal Muscle AMPK and SIRT Activities

**Figure 6:**
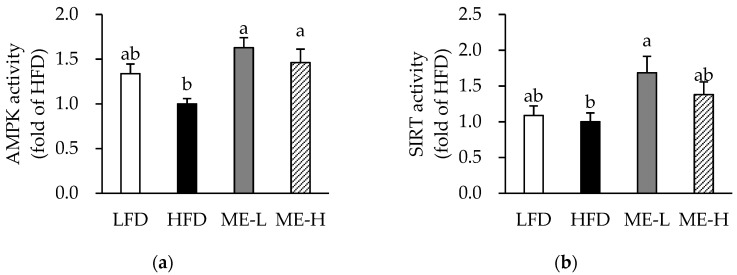
Effect of mulberry fruit extract on AMPK and SIRT activities in skeletal muscle. (**a**) AMPK activity. (**b**) SIRT activity. The protein activities were normalized to total protein concentration. Values are expressed as mean ± SEM (n = 8/group). Bars with different letters indicate a significant difference at *p* < 0.05. Abbreviations: LFD, low-fat diet; HFD, high-fat diet; ME-L, HFD + 5 g/kg diet of mulberry fruit extract; ME-H, HFD + 10 g/kg diet of mulberry fruit extract; AMPK, AMP-activated protein kinase; and SIRT, sirtuin.

The skeletal muscle AMPK activities of the ME-L and ME-H groups were significantly higher compared to that of the HFD group (*p* < 0.05; Figure 6a). In addition, the SIRT activity of the ME-L and ME-H groups was higher than that of the HFD group, and a statistically significant difference was observed in the ME-L group specifically (*p* < 0.05; Figure 6b).

## 4. Discussion

Obesity is closely associated with inflammation, muscle atrophy and impaired mitochondrial biogenesis, and metabolic abnormalities [4]. Inflammatory molecules derived from adipose tissue are potent stimulants for the production of ROS and nitrogen, and dysfunctional mitochondria are responsible for the increase in oxidative stress in obese individuals [1]. In a previous study, adiposity was positively correlated with the concentration of biomarkers associated with oxidative stress, while fruit consumption was inversely related to the lipid peroxidation level [1]. Moreover, some studies have reported that mulberry fruit exerts beneficial effects on several risk factors associated with obesity, such as oxidative stress and inflammation [15,16]. Therefore, this study was conducted to investigate the effects of ME on HFD-induced inflammation and mitochondrial function in rats. The present study demonstrated, for the first time, that 14 weeks of ME consumption ameliorated adipose tissue inflammation and skeletal muscle mitochondrial function in HFD-fed rats by modulation of miR-21/132/143 expression and AMPK/SIRT activities, respectively. In the current study, the mulberry fruit extract was prepared by HHP-processing, which has been reported to facilitate the extraction of bioactive compounds from raw materials [19].

Studies have reported that in obese conditions, enlarged adipocytes lose their ability to store energy, resulting in the release of fatty acids and their absorption into the liver [27]. In this study, 14 weeks of ME supplementation tended to reduce body weight and body weight gain without changing the total amount of food intake. The ME also improved HFD-induced dyslipidemia and hepatic lipid accumulation. The doses of ME used in this study were 0.5 and 1.0% (*w/w*), which were determined based on previous studies with mulberry fruit extract [18,28]. At the dose given, the liver weight and serum AST level were not increased, indicating that the rats tolerated the ME well. Instead, the ME alleviated the HFD-induced elevation of serum ALT levels. ALT is suggested to be a more specific indicator of liver damage than other parameters considering the ALT is present primarily in the liver [29]. In general, a slight elevation of aminotransferase is found in non-alcoholic fatty liver disease [29]. Therefore, the ME was thought to alleviate HFD-induced hepatic toxicity partially by reducing hepatic lipid accumulation.

An increase in WAT mass is an essential indicator of obesity, as most of the surplus energy is stored in the WAT as TG. In this study, the HFD increased the relative weight of adipose tissue fat pads and size of adipocytes. In contrast, supplementation with ME alleviated HFD-induced increases in WAT mass and adipocyte size, indicating that the ME is effective in inhibiting obesity, presumably by reducing lipid accumulation in adipocytes. In a similar manner, Song et al. have reported that ethanolic extract of mulberry fruit attenuated HFD-induced body weight gain and adipose tissue weight increase in mice [30]. In addition, purified mulberry anthocyanins also showed anti-obesity properties by reducing epididymal adipose tissue weight in HFD-induced obese mice [31,32]. As the ME alleviated HFD-induced adipocyte hypertrophy, we further analyzed the mRNA expression of adipogenic genes. Adipogenesis is the process by which preadipocytes differentiate into mature adipocytes. Since mature adipocytes have the ability to store fat, inhibiting adipogenesis could be a promising strategy for preventing obesity. PPAR-γ is a type of transcription factor acting on the differentiation and proliferation of preadipocytes [33]. SREBP-1c is a transcription factor contributing to the generation of PPAR-γ ligands and aP2 is a carrier protein for fatty acids that contributes to fat accumulation in mature adipocytes through lipid biosynthesis pathways [33]. This study demonstrated that the ME inhibited the mRNA expression of adipogenic genes including PPAR-γ, SREBP-1c, and aP2 in WAT. Therefore, we speculated that the inhibitory effects of ME on obesity might be partially associated with the reduction of the expression of adipogenic genes.

Enlarged adipocytes stimulate an inflammatory signaling cascade, thereby releasing inflammatory mediators from adipocytes [34]. NF-κB is a transcription factor that promotes gene expression related to the inflammatory responses. Under normal conditions, NF-κB exists in the cytoplasm as an inactive form consisting of p50, p65, and an inhibitor of κBa (IκBa) subunits [35]. In response to the inflammatory stimuli, IκBa is degraded by ubiquitination, which results in the p65 subunit being transferred into the nucleus and stimulating the initiation of the transcription of its target genes such as TNF-α and IL-6 [35]. Notably, several studies have reported that the NF-κB signaling pathway is stimulated in obese adipose tissue [2]. In the present study, the ME lowered the mRNA and protein levels of pro-inflammatory cytokines such as TNF-α, IL-6, and MCP1 in WAT. In addition, the ME decreased the nuclear levels of total NF-κB (p65) and phosphorylated NF-κB (p65). These results are consistent with a previous study, in which the mRNA expression of TNF-α and IL-6 was suppressed in the adipose tissue of obese mice supplemented with mulberry juice [36]. Therefore, it is postulated that the ME effectively alleviates obesity-induced adipose tissue inflammation partially by the inhibition of NF-κB signaling.

Inflammatory mediators secreted from adipocytes increase the permeability of immune cells [25]. Indeed, obese adipose tissue exhibits increased macrophage infiltration [37]. A phenotypic switch from anti-inflammatory M2 macrophages to pro-inflammatory M1 macrophages occurs in the adipose tissues of obese individuals [25]. M1 macrophages express NOS2 (an enzyme catalyzing the production of NO) and secrete high levels of pro-inflammatory cytokines such as TNF-α and IL-6 [25]. In contrast, M2 macrophages are characterized by the expression of arginase (an enzyme that blocks iNOS activity) and the production of anti-inflammatory cytokines [25]. In this study, the ME inhibited the expression levels of F4/80 (a pan-macrophage marker) as well as NOS2, CD68, and CD11c (M1 macrophage markers) in WAT, while increasing the expression levels of ARG1 and CD163 (M2 macrophage markers). In addition, the serum level of NO was reduced by the ME, suggesting that the ME inhibited macrophage infiltration and polarization into the M1 type due to the HFD. Similarly, a previous study has shown that mulberry fruit ethanolic extract inhibited the expression of macrophage-specific markers such as F4/80 and CD64 in the adipose tissue stromal vascular fraction, in addition to TNF-α and MCP1 expression in adipose tissue from adenovirus 36 infection-induced obese mice [17]. Therefore, it demonstrates that the ME could prevent obesity-induced inflammation partially by preventing macrophage infiltration and M1 polarization in adipose tissue.

MicroRNA is a small non-coding RNA consisting of about 22 nucleotides. Recently, it has received much attention from many nutrition researchers because of its regulatory properties of gene expression at the post-transcriptional level [38]. Several miRNAs have been reported to be involved in adipogenesis and inflammation, and a few of them are suggested to be expressed differently in obesity [3]. Notably, miR-21, miR-132, and miR-143 are proposed to be regulated in response to nutrient excess and to control both fat accumulation and inflammation in adipocytes [3]. In mesenchymal stem cells isolated from human adipose tissue, miR-21 expression was up-regulated during adipogenic differentiation [39]. Due to the multi-functional role of miRNAs, miR-21 can also activate inflammatory responses by modulating NF-κB pathways [40]. MiR-132 has also been reported to activate NF-κB upon nutrient excess, thereby inducing IL-6 and MCP1 transcription in primary preadipocytes and adipocytes [3]. In addition, miR-143 is known for accelerating the differentiation of adipocytes [41]. In a previous study by Liang et al., cyanidin-3-glucoside, a major anthocyanin of mulberry fruit, inhibited miR-138 expression in breast cancer cells, resulting in increased SIRT1 mRNA translation [42]. However, there is still little information regarding the regulatory effects of mulberry fruit on miRNAs. Interestingly, in this study, the ME mitigated HFD-induced increases in miR-21, miR-132, and miR-143 expression levels in the WAT. From these data, it is suggested that the inhibitory effect of ME on adipogenesis and inflammation in obese rats might be related to reduced miR-21, miR-132, and miR-143 expression.

Mitochondria are dynamic organelles that regulate cellular metabolism. In skeletal muscle, the metabolic regulation of mitochondria is undoubtedly important due to its higher energy demands [43]. In a previous study, it has been reported that the chronic consumption of a high-fat/high-sucrose diet is linked to a decrease of muscle mitochondrial density measured by electron microscopy and the mtDNA copy number [44]. Moreover, studies have shown that obesity-induced high-circulating lipids and inflammatory mediators lead to mitochondrial dysfunction in skeletal muscle [8,45]. Dysfunctional mitochondria result in oxidative stress and inflammation, directly or indirectly contributing to metabolic problems [46]. Here, we showed that the ME increased the mitochondrial content and mRNA expression levels of SIRT1, PGC-1α, CPT-1β, and UCP3 in skeletal muscle. Mitochondrial DNA content serves as a useful biomarker of mitochondrial mass and function [26]. SIRT1 is a NAD-dependent protein deacetylase that interacts with PGC-1α and contributes to the regulation of energy metabolism [47]. PGC-1α regulates cellular energy homeostasis in part by promoting the transcription of genes related to mitochondrial biogenesis and function [48]. Both CPT-1β and UCP3 are located in the outer membranes of mitochondria. CPT-1β acts as a rate-limiting enzyme in fatty acid oxidation by catalyzing the relocation of the acyl group of a long-chain fatty acyl-CoA to l-carnitine. UCPs catalyze proton leaks from the inner mitochondrial membrane, releasing energy in the form of heat. UCP3 gene expression is localized to muscle and brown adipose tissue, and is up-regulated by environmental stimuli [49]. In a study by Matsukawa et al., cyanidin-3-glucoside, one of the major anthocyanins in mulberry fruit, increased PGC1α expression in the skeletal muscle of mice [50]. Moreover, in a human hepatocyte cell line, cyanidin-3-glucoside increased mitochondrial content and the mRNA expression levels of SIRT1, PGC-1α, and their downstream genes [51]. In a study on mulberry fruit, it has been reported that the ethanolic extract of mulberry fruit increased the mitochondrial number, oxidative phosphorylation (OXPHOS) complex protein expression, and mRNA expression of genes related to mitochondrial biogenesis and fatty acid oxidation in C3H10T1/2 cells during brown adipogenesis [52]. From these results, it is postulated that the ME exerts beneficial effects on skeletal muscle mitochondrial function, contributing to the prevention of obesity-associated metabolic disturbances.

AMPK and SIRT are critical enzymes that sense intracellular energy status and regulate many aspects of metabolism [53]. AMPK is activated when the cellular AMP/ATP ratio increases, while SIRT is stimulated by intracellular NAD [47]. Since both enzymes control each other and share many common target molecules, they exert similar effects on various metabolic processes, such as energy metabolism and mitochondrial function [53]. Specifically, AMPK functions together with SIRT1 to enhance mitochondrial function by increasing and stabilizing PGC-1α [9]. In previous studies, it has been reported that AMPK phosphorylates PGC-1α and subsequently activates SIRT1 to mediate the deacetylation of PGC-1α by SIRT1, while SIRT1 acts as an upstream regulator of AMPK signaling pathways [47]. Several studies have reported that mulberry fruit activates AMPK under physiological conditions. For example, in a study by Ou et al., the hypolipidemic effect of mulberry water extract was associated with the stimulation of AMPK in HepG2 cells [54]. Furthermore, in *db*/*db* diabetic mice, mulberry fruit extract improved hyperglycemia and insulin resistance by stimulating AMPK in the skeletal muscle and liver [55]. Although the effects of the ME on increasing AMPK activity have been partially elucidated, the SIRT regulatory effects of mulberry fruit are still unknown. In the present study, we demonstrated that ME consumption enhanced the phosphorylation of AMPK (an active form) and SIRT activities in the skeletal muscle of rats fed an HFD. These results indicate that the regulatory effects of ME on skeletal muscle mitochondria would presumably be due to the activation of AMPK/SIRT signaling pathways.

## 5. Conclusions

Our findings have demonstrated that mulberry fruit alleviates adipose tissue inflammation by regulating microRNA-21/132/143 expression and increases both the skeletal muscle mitochondrial content and AMPK/SIRT activities in rats fed a high-fat diet. Therefore, it is assumed that ME prepared by HHP and combined with enzyme hydrolysis could be used as a nutraceutical to improve inflammation and mitochondrial dysfunction in obesity.

## Figures and Tables

**Table 1:** Physiological variables.

Variable	LFD	HFD	ME-L	ME-H
Initial body weight (g)	54.53 ± 0.77	55.47 ± 1.15	54.80 ± 2.10	54.56 ± 2.10
Final body weight (g)	517.15 ± 13.25 ^b^	593.80 ± 5.74 ^a^	552.71 ± 24.94 ^ab^	547.92 ± 11.98 ^ab^
Body weight gain (g/14 weeks)	462.62 ± 13.21 ^b^	538.33 ± 6.46 ^a^	497.91 ± 24.79 ^ab^	493.36 ± 12.23 ^ab^
Food intake (g/day)	25.07 ± 0.50 ^a^	22.66 ± 0.39 ^b^	20.53 ± 0.84 ^b^	20.57 ± 0.41 ^b^
Energy intake (kcal/day)	90.24 ± 1.81 ^b^	104.21 ± 1.79 ^a^	94.45 ± 3.87 ^b^	94.62 ± 1.87 ^ab^
Energy efficiency ratio	0.052 ± 0.001	0.053 ± 0.001	0.054 ± 0.001	0.053 ± 0.000
Liver weight (g)	13.23 ± 0.31	15.43 ± 0.65	14.17 ± 0.85	14.04 ± 0.44
Serum AST (IU/L)	49.76 ± 1.46	61.62 ± 7.37	56.24 ± 2.94	49.48 ± 1.88
Serum ALT (IU/L)	9.06 ± 0.17 ^b^	10.49 ± 0.54 ^a^	9.84 ± 0.17 ^ab^	8.76 ± 0.33 ^b^

Values are expressed as mean ± SEM (n = 8/group). Means in a row without a common superscript letter are significantly different at *p* < 0.05. Abbreviations: LFD, low-fat diet; HFD, high-fat diet; ME-L, HFD + 5 g/kg diet of mulberry fruit extract; ME-H, HFD + 10 g/kg diet of mulberry fruit extract; AST, aspartate transaminase; and ALT, alanine transaminase.

**Table 2:** Lipid profiles in serum and liver.

Variable	LFD	HFD	ME-L	ME-H
Serum				
TG (mmol/L)	1.12 ± 0.06 ^a^	1.18 ± 0.05 ^a^	0.61 ± 0.13 ^b^	0.58 ± 0.06 ^b^
TC (mmol/L)	3.56 ± 0.07 ^b^	4.07 ± 0.10 ^a^	3.65 ± 0.07 ^b^	3.59 ± 0.11 ^b^
HDL-C (mmol/L)	2.19 ± 0.01 ^c^	2.25 ± 0.03 ^bc^	2.45 ± 0.07 ^ab^	2.61 ± 0.06 ^a^
LDL-C (mmol/L)	0.86 ± 0.07 ^ab^	1.28 ± 0.10 ^a^	0.92 ± 0.09 ^ab^	0.71 ± 0.16 ^b^
Atherogenic index	0.63 ± 0.03 ^b^	0.81 ± 0.05 ^a^	0.49 ± 0.03 ^bc^	0.38 ± 0.06 ^c^
Liver				
Total lipids (mg/g)	33.76 ± 1.03 ^c^	64.72 ± 5.62 ^a^	52.34 ± 3.71 ^ab^	42.55 ± 3.74 ^bc^
TG (μmol/g)	6.51 ± 0.45 ^c^	17.37 ± 2.19 ^a^	13.31 ± 1.50 ^ab^	10.68 ± 1.38 bc
TC (μmol/g)	4.81 ± 0.36 ^b^	9.07 ± 1.04 ^a^	5.41 ± 0.42 ^b^	4.72 ± 0.53 ^b^

Values are expressed as mean ± SEM (n = 8/group). Means in a row without a common superscript letter are significantly different at *p* < 0.05. Abbreviations: LFD, low-fat diet; HFD, high-fat diet; ME-L, HFD + 5 g/kg diet of mulberry fruit extract; ME-H, HFD + 10 g/kg diet of mulberry fruit extract; TG, triglycerides; TC, total cholesterol; HDL-C, high-density lipoprotein cholesterol; and LDL-C, low-density lipoprotein cholesterol.

## Data Availability

The original contributions presented in the study are included in the article and Supplementary Materials. Further inquiries can be available from the corresponding author upon request.

## Supplementary Materials

The following are available online at https://www.mdpi.com/article/10.3390/antiox10091453/s1, Table S1: the compositions of the experimental diets; and Table S2: primers used for the quantitative reverse transcriptase-polymerase chain reaction.
