# Supplementary material for: Mulberry (Morus alba L.) Fruit Extract Ameliorates Inflammation via Regulating MicroRNA-21/132/143 Expression and Increases the Skeletal Muscle Mitochondrial Content and AMPK/SIRT Activities
Source: Antioxidants (Basel). 2021 Sep 13;10(9):1453. doi: 10.3390/antiox10091453 (PMC8468054; doi:10.3390/antiox10091453)
Supplement: Supplementary file 1 [file antioxidants-10-01453-s001.zip › antioxidants-1341396-supplementary.pdf]

Supplementary Materials

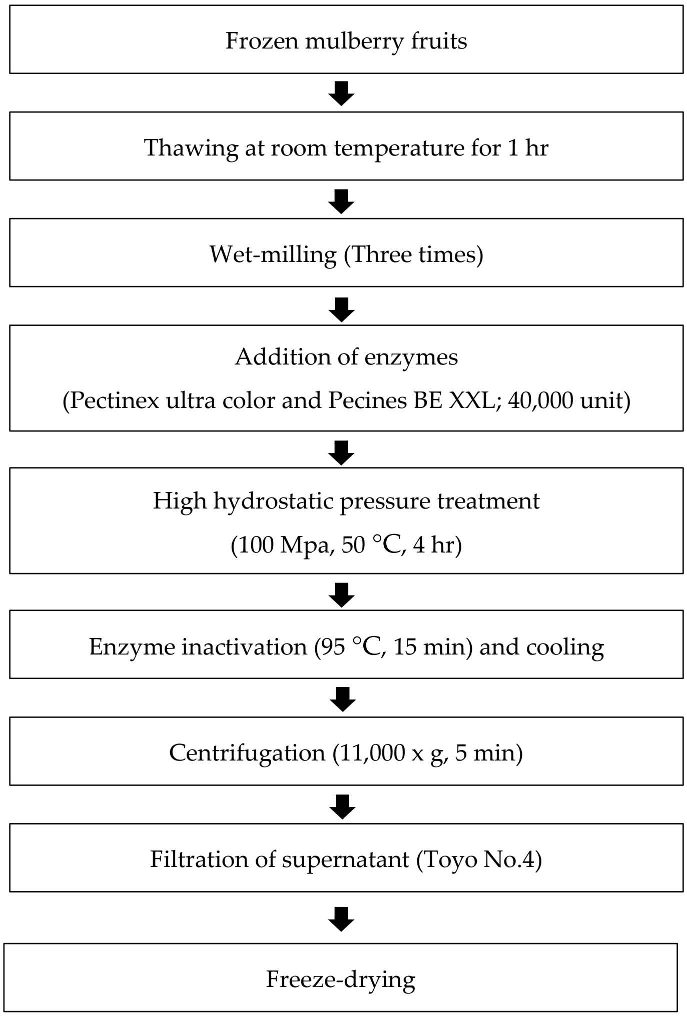

Figure S1. Schematic illustration of the preparation method for mulberry fruit extract (ME).

Table S1. The composition of experimental diets.

Ingredient (g/kg)	LFD [1]	HFD [2]	ME-L	ME-H
Casein	140	170.73	170.73	170.73
L-Cystine	1.8	2.2	2.2	2.2
Corn starch	495.692	201.71	196.71	191.71
Maltodextrin	125	155	155	155
Sucrose	100	121.95	121.95	121.95
Cellulose	50	60.98	60.98	60.98
Soybean oil	40	-	-	-
Lard	-	229.5	229.5	229.5
Mineral mix [3]	35	42.68	42.68	42.68
Vitamin mix [4]	10	12.2	12.2	12.2
Choline bitartrate	2.5	3.05	3.05	3.05
ME	-	-	5	10
Total	1,000	1,000	1,000	1,000
Fat (Kcal %)	10	45	45	45
Energy (Kcal/g)	3.6	4.6	4.6	4.6

[1] AIN-93G purified rodent diet with slight modification.

[2] Modified AIN-93G purified rodent diet with 45% energy from lard.

[3] AIN-93G mineral mix.

[4] AIN-93 vitamin mix.

LFD, Low-fat diet; HFD, high-fat diet; ME-L, HFD + 5 g/kg diet of high hydrostatic pressure extract of mulberry fruit; ME-H, HFD + 10 g/kg diet of high hydrostatic pressure extract of mulberry fruit.

Table S2. Primers used for quantitative reverse-transcriptase polymerase chain reaction.

Gene	Accession number	Primer sequences (5′- 3′)	Product size (bp)
β-actin	NM_031144	F: GGCACCACACTTTCTACAAT<br>R: AGGTCTCAAACATGATCTGG	123
PPARγ	NM_001145366	F: TGTGGGGATAAAGCATCAGG<br>R: CAAGGCACTTCTGAAACCGA	175
STEBP-1c	AF286470.2	F: AGGAGGCCATCTTGTTGCTT<br>R: GTTTTGACCCTTAGGGCAGC	134
aP2	NM_053365	F: TCACCCCAGATGACAGGAAA<br>R: CATGACACATTCCACCACCA	140
F4/80	NM_001007557	F: GATTGGTCCCTTGGCAAGCA<br>R: ATCTCGTACCTGGCGGTTGA	109
NOS2	XM_006246949	F: TCCTGCCACCTTGGAGTTCA<br>R: TGGTCACCTCCAGCACAAGA	152
CD68	NM_001031638	F: ATCATTGGCCTGGTCCTCCTG<br>R: GGGCTGGTAGGTTGATTGTCG	87
CD11c	XM_006230382	F: CAGAACCCGTCCACCCAATG<br>R: GATGTCACAGCGGAAGTGCA	129
ARG1	NM_017134	F: ACATCGGCTTGCGAGATGTG<br>R: GCCAATTCCCAGCTTGTCCA	101
CD163	XM_017592651	F: GCTGGCGTGACATGTTCTGA<br>R: AAACCACGTCGGCATCTGTC	151
TNF-α	NM_012675	F: CCCCTTTATCGTCTACTCCT<br>R: ACTACTTCAGCGTCTCGTGT	139
IL-6	NM_012589	F: ATAGTCCTTCCTACCCCAAC<br>R: TGCCGAGTAGACCTCATAGT	143
MCP1	NM_031530	F: ACTCACCTGCTGCTACTCAT<br>R: CTACAGCTTCTTTGGGACAC	101
SIRT1	XM_008772947	F: AGGGAACCTCTGCCTCATCT<br>R: GAGGTGTTGGTGGCAACTCT	199
PGC1-α	NM_031347	F: GCACCAGAAAACAGCTCCAA<br>R: TTACTGAAGTTGCCATCCCG	130
CPT-1β	NM_013200	F: TGTACTAGCGAGTCCACGGC<br>R: GGTGTTTTTCGGAGGCTTTC	100
UCP3	NM_013167	F: CAGTGACCTGTGCTCAACCC<br>R: CCACAGTCCCCTGACTCCTT	146

PPAR-γ, peroxisome proliferator-activated receptor-γ; SREBP-1c, sterol regulatory element-binding protein-1c; aP2, adipocyte protein 2; NOS2, nitric oxide synthase 2; ARG1, arginase 1; CD, cluster of differentiation; TNF-α, tumor necrosis factor-α; IL-6, interleukin 6; MCP1, monocyte chemoattractant protein 1; SIRT1, sirtuin 1; PGC-1α, peroxisome proliferator-activated receptor gamma coactivator 1α; CPT-1β, carnitine palmitoyltransferase 1β; UCP3, uncoupling protein 3.
